# High Neutrophil-to-Lymphocyte Ratio and Platelet-to-Lymphocyte Ratio Are Associated with Poor Survival in Patients with Hemodialysis

**DOI:** 10.1155/2021/9958081

**Published:** 2021-05-19

**Authors:** Jialing Zhang, Xiangxue Lu, Shixiang Wang, Han Li

**Affiliations:** Department of Blood Purification, Beijing Chao-Yang Hospital, Capital Medical University, Beijing 100020, China

## Abstract

**Background:**

The neutrophil-to-lymphocyte ratio (NLR) and platelet-to-lymphocyte ratio (PLR) are markers for systemic inflammation condition. Although NLR has emerged as a risk factor for poor survival in end-stage renal disease (ESRD) patients, the relationship between PLR and mortality is still unknown. We aimed to explore the interaction of NLR and PLR in predicting mortality in hemodialysis (HD) patients.

**Method:**

We enrolled 360 HD patients for a 71-month follow-up. The endpoint was all-cause and cardiovascular (CV) mortality. Pearson correlation analysis was conducted to evaluate the relationship between factors and NLR or PLR. Kaplan-Meier curves and Cox proportional analysis were used to assess the prognostic value of NLR and PLR.

**Results:**

NLR was positively correlated with neutrophil and negatively correlated with lymphocyte, hemoglobin, and serum albumin. PLR was positively correlated with neutrophil and platelet and negatively correlated with lymphocyte and hemoglobin. In multivariate Cox regression, a higher NLR level was independently associated with all-cause mortality (OR 2.011, 95% CI 1.082-3.74, *p* = 0.027), while a higher PLR level might predict CV mortality (OR 2.768, 95% CI 1.147-6.677, *p* = 0.023) in HD patients.

**Conclusion:**

NLR and PLR are cheap and reliable biomarkers for all-cause and CV mortality to predict survival in HD patients.

## 1. Introduction

Chronic kidney disease (CKD) is defined by the presence of kidney damage or continuous decreased kidney function for three or more months. CKD is highly prevalent in the world with a high risk of mortality and morbidity. Cardiovascular disease (CVD) is considered as the main cause of death in end-stage renal disease (ESRD) patients [1]. Patients with CKD tend to have elevated levels of inflammatory mediators, probably owing to excessive oxidative stress and extracellular fluid overload [2]. Several traditional inflammatory cytokines, such as C-reactive protein (CRP), interleukin-6, and tumor necrosis factor-*α*, are inversely associated with kidney function and positively with poor survival [3, 4].

The neutrophil-to-lymphocyte ratio (NLR) can easily be calculated by the ratio of neutrophils to lymphocytes in peripheral blood, while the platelet-to-lymphocyte ratio (PLR) is obtained by dividing the absolute platelet count by the absolute lymphocyte count. NLR and PLR were regarded as novel markers of inflammation in ESRD patients [5, 6]. Our previous studies have proved NLR to be a risk factor for arterial stiffness, CV, and all-cause mortality in peritoneal dialysis (PD) and hemodialysis (HD) patients [7, 8]. Platelets play a key role in the process of liver inflammation. Several platelet indices, such as platelet count, mean platelet volume, and platelet distribution width, could predict liver fibrosis [9, 10]. However, the relationship between PLR and mortality in CKD patients was still limited and controversial [11, 12].

In this study, we would like to explore the effect of the combination of NLR and PLR on mortality in maintenance HD patients.

## 2. Methods

### 2.1. Study Design and Subjects

A total of 360 ESRD patients who commenced HD in the Department of Blood Purification, Beijing Chao-Yang Hospital, Capital Medical University, between January 2015 and January 2021 were recruited into the study. The inclusion criteria included ESRD patients having no residual renal function and having undergone regular HD (three times/week for 4 h/session with standard bicarbonate dialysate) treatment for at least 3 months. Patients with any heart failure; a recent acute coronary or cerebrovascular event; autoimmune disease; malignancy; liver cirrhosis according to clinical and biochemical data, as well as imaging examination (computed tomography or B-ultrasound) or active infection at the time of commencement of HD; and medication history of aspirin, statins, steroids, or immunosuppressive drugs were excluded from this study.

All patients were followed for 71 months. The primary endpoints were all-cause mortality and CV mortality. CV death included death caused by myocardial infarction, heart failure, cardiac arrest, cerebrovascular accident, or peripheral vascular disease [13]. We classified the patients into four groups according to the median of NLR and PLR. All patients provided written informed consent to the protocol, which was approved by the ethics committee of Beijing Chao-Yang Hospital, Capital Medical University.

### 2.2. Clinical and Biochemical Measurements

Blood samples were obtained from each patient early in the morning after a 10 h overnight fast before initiation of the midweek HD session and were analyzed for complete blood cell count, differential leukocyte count, total cholesterol, triglyceride (TG), CRP, hemoglobin (Hb), serum albumin (ALB), and serum calcium and serum phosphorus levels. Blood chemistry parameters were assayed by standardized and automated techniques in the same laboratory. NLR was calculated as the ratio of neutrophils to lymphocytes while PLR was calculated from the differential count by dividing the absolute neutrophil count by the absolute lymphocyte count.

### 2.3. Statistical Analysis

All data were analyzed using IBM SPSS software, version 23.0 for Windows. Categorical data are expressed as the number (%). Continuous data were reported as the mean ± standard deviation (±SD) or median and interquartile range depending on their distribution. The Kruskal-Wallis test was used for quantitative variables and the chi-squared test for categorical variables among groups. Pearson analysis was performed between related factors and NLR and PLR. Survival curves were estimated by Kaplan-Meier analysis and compared by the log-rank test. We investigated the prognostic value of NLR and PLR for all-cause and CV mortality by Cox proportional analysis. The odds ratios (ORs) and the 95% confidence intervals (CIs) were calculated for each group. *P* values < 0.05 were considered statistically significant.

## 3. Results

### 3.1. Clinical Characteristics of the Study Population

The patients were 60 (52-69) years of age; 55.8% were male. The median of neutrophil, platelet, and lymphocyte counts was 4.05 (3.18‐5.01) × 10^9^/l, 171.5 (134‐216) × 10^9^/l, and 1.17 (0.9‐1.5) × 10^9^/l, respectively. Median NLR and PLR were 3.42 and 142.38. The cohort was categorized into four groups (Group A, NLR ≤ 3.42 and PLR ≤ 142.38; Group B, NLR ≤ 3.42 and PLR > 142.38; Group C, NLR > 3.42 and PLR ≤ 142.38; and Group D, NLR > 3.42 and PLR > 142.38). The level of neutrophil, lymphocyte, platelet, Hb, NLR, and PLR was significantly different among the four groups as shown in Table 1.

### 3.2. Relationship between Relevant Clinical Factors and NLR and PLR

As shown in Table 2, NLR was positively correlated with neutrophil count (*r* = 0.625, *p* < 0.001) and negatively correlated with lymphocyte count (*r* = −0.457, *p* < 0.001), Hb (*r* = −0.196, *p* < 0.001), and serum ALB (*r* = −0.184, *p* < 0.001). PLR was positively correlated with neutrophil count (*r* = 0.152, *p* = 0.004) and platelet count (*r* = 0.473, *p* < 0.001) and negatively correlated with lymphocyte count (*r* = −0.543, *p* < 0.001) and Hb level (*r* = −0.219, *p* < 0.001).

### 3.3. The Interaction between NLR and PLR in Predicting All-Cause and CV Mortality

**Figure 1:**
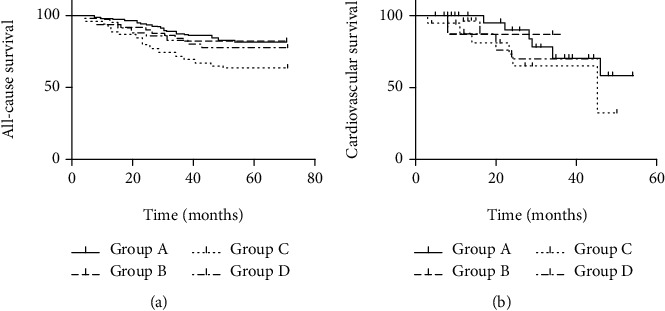
Kaplan-Meier plots for all-cause and cardiovascular mortality according to the groups in hemodialysis patients. (a) Kaplan-Meier plots for all-cause mortality according to the groups in hemodialysis patients (log-rank test, *p* = 0.035). (b) Kaplan-Meier plots for cardiovascular mortality according to the groups in hemodialysis patients (log-rank test, *p* = 0.041).

By January 2021, the follow-up period was 71 months. During this period of follow-up, 81 (22.5%) patients died and 62 deaths were of CV causes. There was a significant difference in Kaplan-Meier survival curves for all-cause and CV mortality among patients in different groups (log-rank test, *p* = 0.035 and *p* = 0.041) as shown in Figure 1.  Table 3 performs the unadjusted and adjusted ORs of Cox proportional analysis for the mortality among four groups. We only found a significant relationship between Group C and all-cause mortality when comparing to Group A. On the other hand, the significant difference between NLR and PLR and CV mortality was only found in Group B compared to Group A in the adjusted Cox regression analysis.

## 4. Discussion

In this study, we investigated the effect of the interaction between NLR and PLR on all-cause and CV mortality in maintenance HD patients. Pearson's analysis showed that NLR and PLR were negatively correlated with the level of ALB and Hb. In addition, we found that a higher level of NLR was seemed to be a risk factor for all-cause mortality, while an elevated level of PLR might serve as a more effective predictor for CV mortality than NLR in HD patients.

CKD is an increasing public health concern around the world with a high risk for CVD, including coronary artery disease, myocardial infarction, and stroke. It is critical to definite prognostic factors for CKD patients to improve survival in the clinical practice. Low-grade inflammation might play a key role in the progression of chronic diseases, such as diabetes, atherosclerotic CVD, and CKD [14]. A prior large cohort study including 44,114 ESRD patients receiving HD has performed that an increased neutrophil count and decreased lymphocyte count were independent predictors for mortality [15]. NLR and PLR can be easily calculated from the routine blood cell count and are more stable and predictive than each parameter alone. Similar to NLR, PLR served as a novel marker of systemic inflammation, and both biomarkers were shown to be independently related to other inflammatory markers [16] and composite adverse outcomes in various diseases, such as chronic obstructive pulmonary disease [17], metastatic renal cell carcinoma [18], non-small-cell lung cancer [19], and cervical cancer [20]. Furthermore, NLR and PLR could also predict the presence of proteinuria [21].

In this study, we found that NLR and PLR were negatively related to the concentration of Hb and serum ALB in maintenance HD patients, which could reflect the nutritional status, relatively. Inflammation status might inhibit the progress of ALB synthesis [22]. And a poor nutritional condition might increase the risk of adverse renal outcomes in advanced CKD patients. CRP, a widely accepted biomarker of inflammation, was positively related to an elevated value of NLR and PLR [23]. However, we did not find any significant relationship between neither NLR nor PLR and CRP in our HD populations.

In this study, we only found that a higher level of NLR, but not PLR, was significantly related to a higher risk of all-cause mortality in HD patients. Furthermore, the elevated level of PLR was better than NLR to predict CV mortality. Previous studies have confirmed the prognostic value of NLR for all-cause and CV mortality in both nondialytic cohorts, HD and PD patients [12, 24–27]. In the Cox multivariate analysis adjusted for other confounding factors, we found that only HD patients in Group C with a high level of NLR (NLR > 3.42) and a low level of PLR (PLR ≤ 142.38) suffered from a higher risk of all-cause mortality. Prior studies regarded PLR ≥ 118.53 and PLR > 130.4 as predictors for CV mortality which were relatively better than NLR in HD and PD patients [11, 28]. However, Tatar et al. [12] showed that PLR was not associated with neither all-cause mortality nor requirement of renal replacement therapy in stage 3-5 CKD patients over the age of 65. In a word, the prognostic value of PLR on CV mortality among ESRD patients was still unknown. Our study included more ESRD patients undergoing HD than before, and the duration of follow-up was relatively longer. The findings showed that an NLR > 3.42 and PLR > 142.38 were associated with increasing CV mortality.

Inflammation was a key component of the malnutrition-inflammation-atherosclerosis and calcification syndrome (MIAC syndrome) which might aggravate atherosclerotic CVD. A higher NLR value could independently predict endothelial dysfunction and poor survival in CKD [25], peripheral arterial occlusive disease [29] and patients undergoing coronary artery bypass grafting [30]. Moreover, activated neutrophils could increase the secretion of myeloperoxidase, matrix metalloproteinase-2, matrix metalloproteinase-9, and reactive oxygen metabolites [31]. These mediators could stick to endothelium and promote early atherosclerosis development and plaque destabilization [32]. On the other hand, neutrophil extracellular traps, including circulating cell-free DNA, are released by apoptotic neutrophil. They could lead to an increasing risk for mortality and inflammation status [33, 34]. After the Cox multivariate analysis adjusted for age, sex, lymphocyte count, Hb, and serum ALB, only patients in Group B of the low level of NLR (NLR ≤ 3.42) and high level of PLR (PLR > 142.38) were companied with a higher risk for CV mortality in this study. The elevated level of PLR might represent a condition of platelet overactivation and lymphopenia. Lymphopenia seemed to play a role in ischemia or reperfusion injury [35]. The progression of CKD was also proved to be associated with a prothrombotic status and changes in platelet function [36]. The effect of the combination of NLR and PLR on CV mortality might be modified by the value of NLR. The definite pathogenesis of PLR on all-cause and CV mortality should be explored further by more fundamental research.

The present study had some limitations. First, the study subjects were included in a Chinese single center which might lead to selection bias. Second, we did not compare NLR or PLR with other inflammatory markers, such as interleukin-6 and tumor necrosis factor-*α*, which might interfere with the prognostic value of NLR and PLR.

## 5. Conclusion

NLR and PLR were inexpensive and convenient biomarkers for inflammation. This study demonstrated that a high NLR value was associated with an increased risk of all-cause mortality, while PLR emerged as a better risk factor for CV mortality in maintenance HD patients.

## Figures and Tables

**Table 1:** Demographic and clinical characteristics of the study patients.

	Total	Group A	Group B	Group C	Group D	*p*
Age (year)	60 (52-69)	59.43 ± 14.19	60.12 ± 15.61	62.86 ± 13.31	59.76 ± 11.7	0.487
Male (%)	55.8	52.7	56	53.1	60.5	0.614
Neutrophil (10^9^/l)	4.05 (3.18-5.01)	3.72 ± 1.3	3.02 (2.54-3.66)	4.69 (3.94-6)	4.68 (3.74-5.95)	<0.001^∗^
Lymphocyte (10^9^/l)	1.17 (0.9-1.5)	1.59 ± 0.51	1.19 ± 0.35	1.15 ± 0.34	0.93 ± 0.32	<0.001^∗^
Platelet (10^9^/l)	171.5 (134-216)	159.66 ± 53.75	211.54 ± 61.67	134.27 ± 47.39	201.16 ± 66.98	<0.001^∗^
Hb (g/l)	112 (102-121)	116 (108-123)	108.68 ± 15.37	110.49 ± 12.97	110 (98-116.5)	<0.001^∗^
ALB (g/l)	40.55 (38-42.6)	41.4 (38.5-43.2)	40.2 (37.83-41.7)	40.1 (37.2-42.55)	40.2 (38.05-42.55)	0.138
Cholesterol (mmol/l)	4.01 ± 0.95	4.03 ± 0.98	4.16 ± 0.9	3.89 ± 0.94	3.83 (3.3-4.6)	0.417
TG (mmol/l)	1.45 (1.02-2.18)	1.64 (1.11-2.25)	1.37 (1.02-1.93)	1.53 (1.09-2.19)	1.32 (0.96-2.25)	0.109
Ca (mmol/l)	2.28 ± 0.22	2.27 ± 0.24	2.31 ± 0.18	2.22 ± 0.24	2.29 ± 0.2	0.433
P (mmol/l)	1.99 (1.61-2.3)	2.01 ± 0.57	1.89 ± 0.39	1.91 ± 0.62	2.05 ± 0.56	0.37
CRP (mmol/l)	3.55 (1.8-10.28)	2.99 (1.77-9.78)	3.13 (1.37-8.16)	4.87 (2–11.53)	3.78 (1.85-11.74)	0.445
NLR	3.42 (2.54-4.78)	2.39 ± 0.59	2.77 ± 0.48	4.03 (3.65-5.01)	5.09 (4.07-6.62)	<0.001^∗^
PLR	142.38 (110.48-194.55)	102.94 ± 26	167.03 (154.16-190.61)	120.66 (105.81-133.83)	206.01 (168.67-259.43)	<0.001^∗^

^∗^Significant difference according to four groups, *p* < 0.05. Values are means ± SD or median (25th-75th percentile), unless specified otherwise. NLR: neutrophil-to-lymphocyte ratio; PLR: platelet-to-lymphocyte ratio; ALB: albumin; Hb: hemoglobin; TG: triglyceride; Ca: calcium; P: phosphorus; CRP: C-reactive protein.

**Table 2:** Correlation between related variables and NLR and PLR.

	NLR		PLR	
	*r*	*p*	*r*	*p*
Age	0.035	0.506	-0.012	0.822
Neutrophil	0.625	<0.001^∗^	0.152	0.004^∗^
Lymphocyte	-0.457	<0.001^∗^	-0.543	<0.001^∗^
Platelet	0.079	0.136	0.473	<0.001^∗^
Hb	-0.196	<0.001^∗^	-0.219	<0.001^∗^
ALB	-0.184	<0.001^∗^	-0.095	0.071
CRP	0.098	0.063	-0.037	0.485

^∗^Significant difference according to four groups, *p* < 0.05. NLR: neutrophil-to-lymphocyte ratio; PLR: platelet-to-lymphocyte ratio; ALB: albumin; Hb: hemoglobin; CRP: C-reactive protein.

**Table 3:** Univariate and multivariate Cox regression model for all-cause and CV mortality.

	All-cause mortality		CV mortality	
	OR (95% CI)	*p*	OR (95% CI)	*p*
Unadjusted				
Group A	Reference		Reference	
Group B	1.023 (0.476-2.201)	0.954	3.043 (1.267-7.309)	0.013^∗^
Group C	2.329 (1.264-4.292)	0.007^∗^	1.43 (0.678-3.014)	0.347
Group D	1.301 (0.758-2.235)	0.34	1.997 (1.043-3.822)	0.037^∗^
Adjusted				
Group A	Reference		Reference	
Group B	0.641 (0.293-1.402)	0.265	2.768 (1.147-6.677)	0.023^∗^
Group C	2.011 (1.082-3.74)	0.027^∗^	1.099 (0.484-2.491)	0.822
Group D	0.984 (0.569-1.702)	0.954	1.022 (0.393-2.657)	0.964

Adjusted for age, sex, ALB, Hb, and lymphocyte count. ^∗^Significant difference according to four groups, *p* < 0.05. CV: cardiovascular; OR: odds ratio; CI: confidence intervals.

## Data Availability

The datasets generated and analyzed during the current study are available from the corresponding author on reasonable request.
